# Learning by Association in Plants

**DOI:** 10.1038/srep38427

**Published:** 2016-12-02

**Authors:** Monica Gagliano, Vladyslav V. Vyazovskiy, Alexander A. Borbély, Mavra Grimonprez, Martial Depczynski

**Affiliations:** 1 Centre for Evolutionary Biology, School of Animal Biology, University of Western Australia, Crawley, WA 6009, Australia; 2 Department of Physiology, Anatomy and Genetics, University of Oxford, Oxford, OX1 3PT, United Kingdom; 3 Institute of Pharmacology and Toxicology, University of Zurich, Zurich, 8057, Switzerland; 4 Australian Institute of Marine Science, Crawley, WA 6009, Australia; 5 Oceans Institute, University of Western Australia, Crawley, WA 6009, Australia

## Abstract

In complex and ever-changing environments, resources such as food are often scarce and unevenly distributed in space and time. Therefore, utilizing external cues to locate and remember high-quality sources allows more efficient foraging, thus increasing chances for survival. Associations between environmental cues and food are readily formed because of the tangible benefits they confer. While examples of the key role they play in shaping foraging behaviours are widespread in the animal world, the possibility that plants are also able to acquire learned associations to guide their foraging behaviour has never been demonstrated. Here we show that this type of learning occurs in the garden pea, *Pisum sativum.* By using a Y-maze task, we show that the position of a neutral cue, predicting the location of a light source, affected the direction of plant growth. This learned behaviour prevailed over innate phototropism. Notably, learning was successful only when it occurred during the subjective day, suggesting that behavioural performance is regulated by metabolic demands. Our results show that associative learning is an essential component of plant behaviour. We conclude that associative learning represents a universal adaptive mechanism shared by both animals and plants.

The ability to choose among different and often conflicting options, and predict outcomes, is a fundamental aspect of life^1^,^2^,^3^,^4^. One form of choice behaviour is based on establishing an association between an occurrence of external events and the opportunity to satisfy internal homeostatic needs, such as hunger, thirst or sleep. The notion that choices are driven by the expectation of their rewarding outcome goes back to Aristotle^5^ and has been observed extensively across the animal kingdom^6^,^7^,^8^,^9^. However, it remains unknown whether this is also true for plants.

In the complex photosynthetic world of plants, light plays an especially important role in growth and survival. Its role is dual. On the one hand, light energy is necessary for processes of biosynthesis. On the other hand, light provides a time cue for entrainment of the circadian rhythm to the 24-h cycle, thereby optimizing the adjustment of growth and metabolism to the seasonal variation of the photoperiod^10^. Therefore, the ability to detect salient cues that increase efficiency in foraging for light is absolutely essential and confers a significant evolutionary advantage. Plants have recently been found to acquire new behaviours to enhance foraging efficiency for light through the non-associative learning process of habituation^11^, and thus to facilitate photosynthesis and growth. However, it remained unknown whether plants can also learn through forming associations.

To investigate this possibility, we employed a classical conditioning paradigm where a neutral environmental cue (a conditioned stimulus, CS) predicted the occurrence of light, which is biologically significant (an unconditioned stimulus, US). In the first experiment, pea seedlings (n = 45) were entrained to an 8-h light:16-h dark cycle for 5–8 days. In the subsequent 3-d training period, they were kept in darkness with the exception of 1-hour light exposures during the three daily training sessions. Training occurred individually inside a Y-maze, where the airflow produced by a fan ([F] as the CS) and a blue LED light ([L] as the US) were systematically presented according to a specific protocol (Fig. 1 and details in Methods section; see also Extended Data Fig. 1 and Supplementary Video 1).

Prior to training, seedlings were randomly assigned to one of 2 experimental groups. In one group exposure to the light and fan was on the same arm of the maze [F + L], whereas in the other group light and fan were on opposite arms [F *vs* L] (Fig. 1Ai,ii). Accordingly, this design tested for both a positive association of the fan (CS) with light (plant trained to seek out fan as a predictor of light) and a negative association of the fan with light (plant trained to avoid the fan and find light on the side of the tube with no air movement). The protocols were maintained throughout the 3-d training period. However, to render the direction of the incoming light unpredictable, its position with respect to the arm of the maze was re-assigned for each 120-min training session (details in Methods section). During training, the seedlings grew and approached the Y-bifurcation of the maze.

Before the testing day, the seedlings were further subdivided randomly into a test group (n = 26) and a control group (n = 19; the numbers are unequal due to a technical problem). The test group was exposed only to the fan during the three 90-min sessions. In this group, to control for the influence of innate phototropic response, the fan was placed in the arm opposite to last light exposure in the [F + L] group and on the arm of last light exposure in the [F *vs* L] group. The seedlings of the control group were left undisturbed. On the morning after the testing day, we visually inspected the seedlings and recorded the arm of the maze they had grown into (Fig. 1Aiii,iv).

As expected, we found that all seedlings of the control group grew into the arm of the maze where the blue light had been presented in the last training session (white bars; Fig. 2). This result corroborates the well-known innate phototropic response of seedlings to blue light^12^. In contrast, in the test group, the majority of seedlings exhibited a conditioned response to the fan (green bars; Fig. 2). In the [F + L] group, 62% of the seedlings grew towards the fan (Fig. 2A), whereas in the [F *vs* L] group, 69% of the seedlings grew in the direction opposite to the fan (Fig. 2B). Thus, the first experiment has shown that plants are able to form associations to enhance foraging success.

In animals, the circadian system provides a framework for a wide range of behaviours. The ability to anticipate changes in food availability enables efficient interactions of the organism with the environment in a time of day dependent manner, and facilitates learning^13^,^14^. Great progress has been achieved in characterizing the molecular and physiological basis of circadian rhythmicity in plants^15^. However, it remains unknown whether the time of day modulates behavioural processes such as learning, in plants.

In our second experiment, seedlings (n = 83) were trained and tested inside a Y-maze, where temperature and light served as Zeitgebers^16^ (Fig. 1B; details in Methods section). The training and testing procedure corresponded to that of the first experiment. However, exposure to fan and blue light occurred always on the same arm of the maze ([F + L] condition). The main variable was the phase of the 24-h temperature cycle in which the training and testing sessions occurred.

Prior to training, the seedlings were randomly assigned to one of 3 experimental groups. They were maintained under controlled environmental conditions (12-h light:12-h dark coinciding with high-low temperature 21 °C:17 °C). The temperature cycle served as the Zeitgeber that was maintained throughout the training and testing periods. The timing of the experimental ‘day’ (light +21 °C) in the growth chamber varied between the groups (Fig. 3A): Group 1 experienced ‘day’ from 07:00–19:00 (‘Light’), Group 2 from 01:00–13:00 (‘Light-Dark’) and Group 3 from 19:00–07:00 (‘Dark’). After emergence, each seedling was transferred into its individual Y-maze where one of the arms was used to deliver both the unconditioned stimulus (light, US) and the conditioned stimulus (fan, CS). Since both [F vs L] and [F + L] protocols had been equally effective in the first experiment, only the latter protocol was used here. During the three training days, the seedlings were maintained at their respective temperature regimes in total darkness, except for the three 1-h sessions with blue light exposure paired with the fan (see orange and blue rectangular areas; Fig. 3A). These sessions occurred at the same external clock time in the three groups (10:00, 13:00, 16:00), but at different phases of the 24-h temperature cycle. On the testing day, only CS was delivered in the three 90-min sessions (Fig. 1Bii; see orange rectangular areas only, Fig. 3A). The control groups were left undisturbed on the testing day (Fig. 1Biii).

In the ‘Light’ group, the majority of tested seedlings grew towards the fan, opposite to the arm where the light was delivered during the last training session (green bar; Fig. 3Bi), confirming the results of the first experiment. All control seedlings in this experimental group grew towards the arm of the last light exposure (white bar; Fig. 3Bi), also confirming previous findings. However, this was no longer the case in the ‘Light-Dark’ and ‘Dark’ group, in which training and testing occurred at different phases of the temperature cycle. Under these conditions, learning was not successful (47% and 20% in the ‘Light-Dark’ and ‘Dark’ group respectively; green bars; Fig. 3Bii,iii). Growth towards previous light exposure of undisturbed control seedlings was reduced in the ‘Dark’ Group (white and yellow bars; Fig. 3Biii), and abolished in the ‘Light-Dark’ group. In the latter, the innate phototropic response, which is consistently 100% efficient when exposure to light occurs during daylight hours (white bars; Figs 2A,B and 3Bi), is no longer displayed and growth behaviour appears random (white and yellow bars; Fig. 3Bii).

Thus, our results show that pea seedlings develop an association that facilitates growth towards the light based on the occurrence of a neutral cue. This learned behaviour prevails over innate positive tropism to light, which is thought to be the major determinant of growth direction in plants. In both experiments, the ability of seedlings to anticipate both the imminent arrival of light (“when”) and its direction (“where”) based on the presence and position of the fan indicates that plants are able to encode both temporal and spatial information and modify their behaviour under the control of environmental cues. This form of learning is ubiquitous in the animal kingdom^17^,^18^, including all major vertebrate taxa and several invertebrate species^19^ and can also be implemented in artificial networks and machines^20^. Whilst the possibility that plants also learn by association has been considered by earlier studies^21^,^22^, our current study provides the first unequivocal evidence.

The learning paradigm characterised here opens new and exciting opportunities for examining numerous other forms of associative learning mechanisms as well as phenomena that arise within associative learning in plants. For example, our current experimental design could be easily extended by including an experimental condition, where the US precedes (and then overlaps) the CS. If this too elicits the same conditioned response as CS preceding US, this would provide evidence for a form of associative learning known as conditional sensitization. Similarly by extending our design to test different temporal arrangements between the CS and US in learning acquisition (e.g. the CS and US are presented at widely separated intervals and/or in alternate orders), the role of timing and how spatiotemporal relationships between events are encoded within an association in plants could be readily assessed and then compared to findings in animal studies^17^,^23^. Additionally at the ecological level, it would be most interesting to test whether/how plants respond to other, novel neutral stimuli (besides the fan used in our study); this would provide evidence for stimulus generalization, another form of associative learning that occurs when, after a conditioned response has been established to a particular CS, other similar stimuli to the CS will elicit the same response. Because the ability to generalize previous learning and apply it to novel situations endows organisms with the behavioural flexibility to adjust to environmental change without having to learn ‘from scratch’ in each specific situation^24^,^25^, this aspect of associative learning is of great ecological relevance. Accordingly, we believe that experimental efforts directed towards its study under natural conditions could make important contributions to our understanding of plant ecology.

Besides opening up new experimental research avenues, our findings raise some obvious questions about the underlying physiological/molecular mechanisms by which plants can integrate environmental and internal cues and coordinate complex patterns of information during associative learning, so that a more effective, even adaptive, behavioural response can be expressed. At the molecular level, the role of epigenetic reprogramming has been identified as a potential candidate mechanism underlying learning processes across taxa^26^,^27^, including plants^11^,^28^. In multicellular organisms with a nervous system, changes in the synaptic strength between neurons, for example, can be stored as a memory trace that sustain associative learning. In plants and other organisms that do not have a nervous system, modifications of the patterns of interactions between molecules and communication between cells can be stored in a way rather similar to neural networks^29^. Presumably, then, the mechanisms maintaining associative learning operate in plants as in other organisms on the basis of fundamental ‘rules’ that alter the flow of information by modifying the shape and connections within a network via epigenetic changes^26^. While the specific mechanisms that underlie associative learning in plants are to be defined and may be quite different from other system, comparative studies of the molecular tools involved in associative learning across the different systems could be useful in revealing such fundamental ‘rules’. Research in invertebrate animals such as *Aplysia*, for example, represents a remarkable success story and an example of how using a simple behaviour and a non-physiological manipulation resulted in fundamental discoveries going far beyond a specific learning paradigm and species^30^,^31^.

While the mechanisms underlying associative learning in plants remain to be determined, there is little doubt that it is likely to enhance opportunities to locate and capture available light in the environment. It has been shown that auxin signalling systems may be involved in the integration between photo- and mechanosensory information, which enables directional growth to optimize photosynthesis^32^,^33^. Earlier experiments have demonstrated that young plants can form stable memories pertaining to the direction of a light source^34^, while a more recent study showed that plants optimize opportunities to access and forage for light by learning to ignore recurrent, but unimportant stimuli in their environment^11^. Our results extend these findings by showing that plants are able to adapt quickly to changes in the environment and develop anticipatory behaviour, which may play an important role in maintaining metabolic homeostasis.

Consistently, we show in the second experiment that associative learning occurs most readily during the subjective day. This experiment, in which plants were trained at three different time periods within 24 hours, revealed that the learning effect disappears when training occurred during the evening hours when light would not normally be available. This finding is particularly intriguing and bolsters the argument that associative learning is an adaptive response that is only utilized during daylight hours (when it is most useful) via an internal circadian clock. Interestingly, the response of the control group in this experiment was affected by the phase of the temperature cycle. While in the Dark group the majority of seedlings still grew towards the side of the last light exposure, this was not the case for the Light-Dark group. Thus the phototropic response which was 100% efficient in the Light group, proved to be attenuated or abolished in the other groups. Therefore, the main finding of the second experiment is that phototropism of peas is circadian phase-dependent. This could be due to a modulation of light-sensing capacity or of the transmission of light-induced processes to structures affecting growth (see ref. 35 for review of the topic). Given that alignment of the internal circadian phase with external environmental signals is essential to maintain efficient photosynthesis during the day as well as optimal utilization of reserves during the night^36^, plants are likely to incur an energetic cost when performing light-foraging behaviours at a time that is misaligned with the internal circadian signals. Our results suggest that under these conditions, the expression of both innate and learned behaviours is traded off to meet basic metabolic demands in order to ensure growth and survival.

The emergence of associative learning has been proposed as one of the key biological innovations that powered the Cambrian explosion by driving the evolution of new sensory modalities and hence, altering the life and adaptive possibilities of animals^37^,^38^. Our results now show that associative learning is also an essential component of plant behaviour. We propose that the ability to construct, remember and recall new relationships established via associative learning constitutes a universal adaptive mechanism shared by all organisms. The ubiquity of associative learning across taxa, including non-animal groups suggests that the role this learning process plays in nature is thus far underexplored and underappreciated. Our findings raise the possibility that associative learning may have played a similarly important role in the remarkable diversification of the plant kingdom and that this kind of learning emerged in plant and animal groups alike via convergent evolution^39^.

## Methods

### Experiment 1: Germination and growth conditions

All experiments in this study have been performed in the garden pea (*Pisum sativum* cv Massey Gem). Pea seedlings were germinated hydroponically in 250 mL round containers kept in the dark in a 5.3 m^2^ Controlled Environment Room (CER) at the Plant Growth Facilities at the University of Western Australia. First, seeds were soaked in water for 24 hours and then wrapped with clean, wet paper-towel surrounded by an external layer of aluminium foil. Five seeds per roll were used and seed rolls were placed vertically in the round container, immersed in 50 mL of water (replenished daily) and incubated in the dark inside a growth chamber at a constant mean temperature (20 °C ± 0.05 SEM) and humidity (85% ± 0.35 SEM), simultaneously recorded at hourly intervals (U12-011 Temperature/RH Data Logger, HOBO®, Australia). At ca. 09:00 each morning, progress towards germination was evaluated visually in approx. 5–10 sec per seed roll under the dim light of a LED headlamp (with red plastic filter; ca. 0.8 lux). Seeds were considered to have germinated when the radicle was >5 mm long. Ungerminated seeds were discarded. Upon germination, each seedling was planted in soil (Osmocote® seed raising and cutting mix, Scotts Australia) at a depth of 15 mm in the centre of a small black plastic pot (55 mm diameter by 47 mm in depth), which was then sequentially numbered. The soil had been saturated with water and then allowed to drain freely for about 5–10 min prior to planting. Planting occurred in the morning at ca. 09:00 and in the days following planting, plants were watered as needed at this same time. All numbered seedlings were kept in the growth chamber at the same constant temperature and humidity conditions indicated as above, but artificial illumination by cold white fluorescent light was now delivered inside the chamber from the top (at ca. 50 μmol m^−2^ s^−1^ at the soil surface) on a 8-h light:16-h dark photoperiod (09:00–17:00 light period) and for a period of 5 to 8 days (depending on emergence times).

### Y-maze experimental design

**Figure 1:**
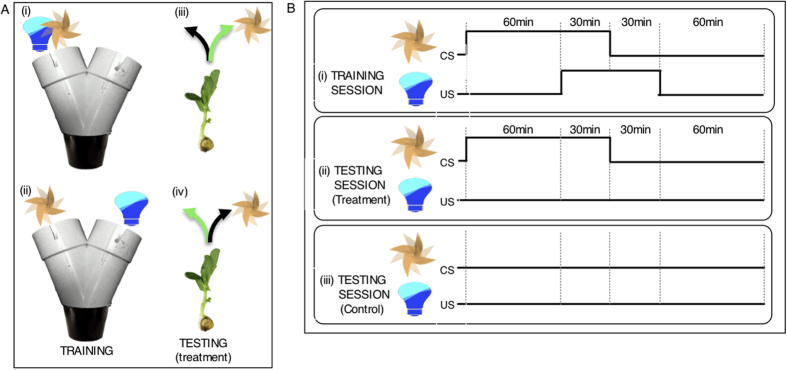
Training and testing protocol for associative learning in pea seedlings. (**A**) During training seedlings were exposed to the fan [F] and light [L] on either the same arm (i) or on the opposite arm (ii) of the Y-maze. The fan served as the conditioned stimulus (CS), light as the unconditioned stimulus (US). During testing with exposure to the fan alone two categories of responses were distinguished. *Correct response*: Seedlings growing into the arm of the maze where the light was “predicted” by the fan to occur [green arrow; iii (corresponding to scenario i) and iv (corresponding to scenario ii)]; *Incorrect response:* Seedlings growing into the arm of the maze where the light was not “predicted” by the fan to occur (black arrow; iii and iv). (**B**) Seedlings received training for three consecutive days before testing. Each training day consisted of three 2-h training sessions separated by 1-h intervals. The 90-min CS preceded the 60-min US by 60 minutes so that there was a 30-min overlap. (i). During the 1-day testing session, seedlings were exposed to the fan alone for three 90-min sessions (ii). Seedlings of the control group were left undisturbed (no fan, no light; iii).

At 09:00 each morning, immediately after light onset, seedlings were checked visually. We recorded the seedlings that emerged out of the soil and were ready to be transferred into their individual custom-designed Y-maze in the morning of the next day to commence training. Each maze unit consisted of a modified 45-degree Y-shaped PVC pipe (dimensions: 55 mm base diameter by 105 mm total height; bifurcation at 65 mm height from base by 95 mm overall width at the bifurcation point; each arm measured 60 mm diameter by 40 mm arm length) fitting on top of the small black plastic pot (Fig. 1A) and secured to a polyurethane foam base. The size of the maze was chosen to allow unrestricted growth inside the maze over a three-day period. Each potted seedling within its maze was allocated to one of the two experimental groups using a simple randomization procedure (i.e. coin flipping; the outcome of each throw was recorded next to a list of consecutive numbers in ascending order, which corresponded to the list of numbered pots where peas were to be planted in upon germination). Subsequently, the plants were kept in complete darkness and at a constant 20 °C ± 0.05 SEM ambient temperature throughout the duration of the study. Special care was taken to not disturb the seedlings during this period, including light exposure or any mechanical stimulation, which prevented us from taking regular measures of the rate of growth.

In classical conditioning, the conditioned stimulus (CS) is initially a neutral stimulus that does not naturally trigger a response from the test subject, while the unconditioned stimulus (US) elicits a natural response. In our experiment, we used blue light as US, and fan as CS. To confirm the well-known tropic response of pea seedlings to blue light^12^ and thus, the suitability of blue light as the US for this study, we performed a pilot experiment, where a group of 10 seedlings was trained and tested inside a Y-maze where a blue LED light (ca. 14 μmol m^−2^ s^−1^ at a 430–505 nm wavelength) was randomly placed on either the left or right arm of the maze and then kept at this fixed position during the entire three-day training and one-day testing phase (Extended Data Fig. 1A). Before 09:00 the morning following the end of the training phase, all seedlings were checked under the dim red-filtered light of the headlamp as described above to ensure that they had not yet grown into one or the other arm. They were also checked to ensure they had grown to be ca. 1 cm away from the bifurcation of the maze and thus, had to choose a direction for their continuing growth during the following day of testing. At 09:00 in the morning after the testing day, all seedlings were inspected and their position in the maze recorded. All 10 seedlings directed their growth toward the arm of the maze where the light was last presented. Similarly, the airflow produced by a small plastic DC12v mini 9-blade fan (35 mm diameter by 10 mm in depth; speed: 5000 rpm; noise level: 11–15 dBA) was examined as a possible candidate CS.

To test whether the fan itself was a behaviourally neutral cue to pea seedlings, we performed a further pilot experiment, where a group of 30 seedlings was trained and tested inside a Y-maze where a blue light was positioned on each arm of the maze, while a fan was randomly placed on one arm of the maze and then kept at this fixed position during an entire training and testing phase (Extended Data Fig. 1B). The presence of the fan had no significant effect on seedlings’ direction of growth when light was presented at both ends of the maze simultaneously (Fisher’s Exact Test, P = 0.796, n = 30).

As described above, all seedlings were inspected at the end of the training phase and their position in the maze was recorded in the morning after the testing day. The exact distance of the seedlings from the bifurcation before the testing day was not recorded. Even though this would have been a desirable measurement to record and include as a co-variable, performing it would have introduced the confounding effects of additional stimuli to our experimental design, which would have changed the entire learning paradigm, and made the results difficult to interpret.

The scoring/recording was carried out by two observers using a blind procedure: one observer would score a seedling’s choice and then read out its number, the other would record the score in the logbook by entering the “choice” next to the number and treatment information regarding that seedling. To avoid any observer bias, the observer who had setup the testing conditions on the previous day always recorded, while the other observer always scored.

### Experiment 1: Training and testing conditions

Seedlings (n = 45) were kept inside their individual Y-maze at constant temperature and in darkness throughout the experiment, except for the time of blue light exposure. They were randomly assigned to either the condition where fan and light were presented on the same arm of the maze [F + L group] or the condition where they were on opposite arms [F *vs* L group] (Fig. 1Ai,ii). In both experimental groups, the conditioning protocol used to test the associative learning abilities of seedlings consisted of three training days with three training sessions (starting at 09:00, 12:00 and 15:00 respectively) per day, and a subsequent testing day. We employed a Forward Delay Conditioning paradigm, whereby during each training session, the fan as the CS always preceded the blue light as the US by one hour and overlapped with it for 30 min (Fig. 1B). Fan and light were always presented in the same position relative to each other according to their assigned group, [F + L] or [F *vs* L]. Their position in each maze, however, was re-assigned for each training session to ensure that the direction of the incoming light was unpredictable. In a [F + L] maze, for example, fan and light were positioned on the right arm in one training session and then together moved to the left arm for the next session.

To ensure that each pea was exposed to the blue light in an equal number of ‘right’ and ‘left’ sides prior to testing, all peas were trained according to the following pattern: day 1, L/R/L; day 2, L/R/R; day 3, R/L/L and then tested on the right. Each training day consisted of three 2-h training sessions separated by 1-h intervals, during which all seedlings were kept in darkness and undisturbed in their individual mazes (Fig. 1Bi). At the end of the 3-d training phase the seedlings were within ca. 1 cm of the bifurcation of the maze and thus, had to choose a direction for their continuing growth during the following day of testing. At this time, seedlings within each experimental group were assigned to either a test group (Fig. 1Bii) or a control group (Fig. 1Biii) using a simple randomization procedure as described above. In the control group (CTR[F + L], n = 10; CTR[F *vs* L], n = 9), the seedlings were kept undisturbed in darkness. The seedlings of the test group (n = 13 per experimental group) were also kept in darkness, but exposed to the fan for three 90-min sessions (starting at 09:00, 12:00 and 15:00 respectively) (Extended Data Fig. 1B). Throughout the three sessions, the fan was positioned either on the same ([F + L]) or the opposite arm of the maze ([F *vs* L]), where the pea had been exposed to light during its last training session (Fig. 1A). This was done to ensure that a possible conditioned response was not confounded by the innate phototropic response to grow into the side of the maze where the light had been presented on the last training session. At 09:00 the morning after the testing day, we inspected the seedlings and recorded the arm of the maze they had grown into. Once scored, seedlings were transferred from the controlled-environment room, transplanted into larger pots and allowed to grow in a glasshouse environment until ready to be replanted in a community garden.

### Experiment 2: Germination and growth conditions

**Figure 3:**
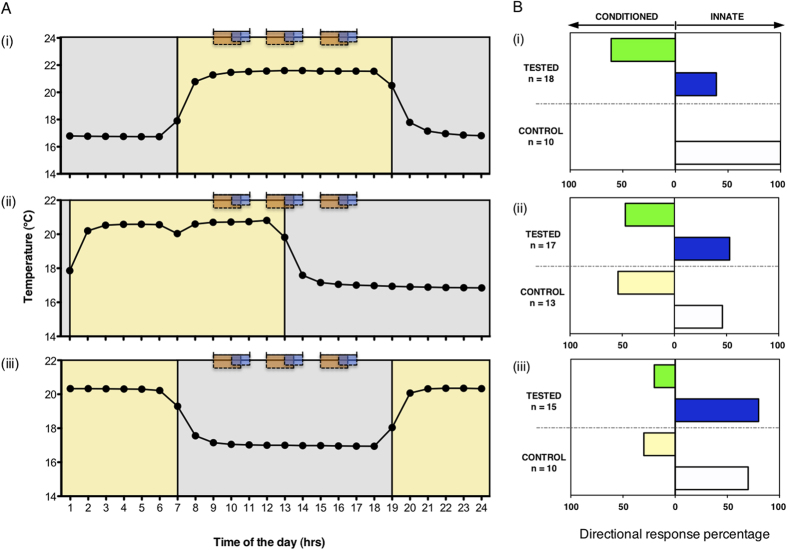
Circadian effects on behavioural performance of pea seedlings. (**A**) Seedlings were kept in incubation chambers, where initially both light and temperature were used as Zeitgebers (temperature = dotted line; mean values across all days; light:dark cycles, yellow:grey shaded areas). During three training days, the seedlings were kept in darkness with the exception of the three training sessions, while the temperature cycle was maintained (note: the LD cycle was not maintained during training). The training (orange and blue rectangular areas, indicating the time of exposure to the fan and the blue light respectively) and testing sessions occurred during the former light phase in the Light group (i), and partly or entirely outside the former light phase in Light-Dark (ii) and Dark group (iii), respectively. (**B**) In the ‘Light’ group (i), the growth response of tested seedlings was significantly different from control seedlings (Two-tailed Fisher’s Exact Test, P = 0.002). All control seedlings grew to the arm of the maze where the blue light had been delivered on the last training day [white bar; (i)], while 61% of tested seedlings grew towards the arm where the fan predicted the blue light to occur [green bar; (i)]. A minority of tested plants (39%) did not form an association [blue bar; (i)]. Under phase-shifted conditions, the tested seedlings did not differ from controls [Two-tailed Fisher’s Exact Test, P = 0.769 for the Light-Dark group (ii); P = 0.653 for the Dark group (iii)]. Phase-shift disrupted the phototropic response of control seedlings [white bars; (ii, iii)], causing only 46% of individuals in the Light-Dark group and 70% in the Dark group to direct their growth towards the side of last light exposure [white bars; (ii, iii)].

In this experiment, dry seeds were first randomly assigned to one of three experimental groups (Light, Light-Dark and Dark, see below). Seeds were initially soaked in water for 24 hours in 250 mL round containers and kept on heated propagation trays. Subsequently, each tray, equipped with a thermostat set at the treatment-specific temperature cycles (recorded at hourly intervals using a HOBO data-logger), was individually housed inside a dark incubation chamber. All experimental groups were maintained at a 12:12 h cycle of ambient temperature (21 °C ± 0.3 SEM during hours 07:00–19:00 (day) and 17 °C ± 0.02 SEM during hours 19:00–07:00 (night). The three experimental groups differed with respect to the timing of their day-night cycle in the chamber relative to external clock time. Specifically, while the 12-h ‘day’ was scheduled from 07:00–19:00 in the first group (Light), it was shifted by 6 hours in the Light-Dark group (01:00–13:00 day: 13:00–01:00 night) and by 12 hours in the Dark group (19:00–07:00 day: 07:00–19:00 night); see yellow and grey colour shaded areas (Fig. 3A).

Following the 24-h soaking, seeds were incubated in paper and allowed to germinate hydroponically (as described above) in the dark chambers at their treatment-specific temperature cycles. As in the first experiment, each germinated seedling was planted in wet soil in a small individually numbered plastic pot to a depth of 15 mm. After planting, seedlings from each experimental group were kept in a growth chamber at their specific light:dark/temperature cycle. During this phase, they also received white light illumination delivered inside the chamber from the top as in the learning experiment, but on a 12-h light:12-h dark photoperiod (see yellow:grey shaded areas, Fig. 3A) synchronized with their group-specific light:dark temperature cycle. The entrainment lasted 5–8 days. Each morning between 06:45 and 07:15, we recorded the seedlings that emerged out of the soil and were ready to be transferred into their individual Y-maze to start training the subsequent morning. To avoid possible confounding effects of changes in light conditions, seedlings in the Dark group were always checked prior to the end of their ‘day’ at 07:00, while those in the Light group were always checked after 07:00, which marked the beginning of their ‘day’.

Each potted seedling within its maze was transferred to a propagation tray heated according to the treatment-specific temperature cycle and kept in darkness for the remainder of the experiment. The temperature cycle served as the Zeitgeber that was maintained throughout the training and testing periods.

### Experiment 2: Training and testing conditions

A total of 83 seedlings (Light, n = 28; Light-Dark, n = 30; Dark, n = 25) were individually trained inside a Y-maze, where the fan [F] and the blue LED light [L] were always positioned together on one of the arms of the maze (as in the [F + L] group of the first experiment). The conditioning protocol was the same as that used in the first experiment. It consisted of three training days of three training sessions per 24 hours, and a subsequent testing day, all delivered between 09:00 and 17:00. Accordingly in this experiment, seedlings received their training and tested sessions at times based on their entrained temperature and photoperiod cycles and corresponding to their “subjective” day (Light group), or were partly (Light-Dark group) or entirely outside their subjective day (Extended Data Fig. 2). For example, plants from the Light group were trained and tested at a time of day matching their entrained light period, while plants from the Dark group were trained and tested at a time of day, which corresponded to their entrained dark period.

During each training session, the fan exposure as the CS always preceded by 1 hour and overlapped by 30 min with the delivery of blue light as the US (Fig. 1Bi). Fan and light were always presented in the same position relative to each other, but their position in each maze was re-assigned for each training session to ensure the source of the light remained unpredictable. Additionally to ensure that each pea was exposed to the blue light in an equal number of ‘right’ and ‘left’ sides prior to testing, we used a simple randomization procedure (as described above) to assign individual peas to one of two alternative 3-day training schedules. Schedule A always started on the right arm and followed the pattern: day 1, R/R/L; day 2, R/L/L; day 3, R/L/R (and tested on the L). Schedule B always started on the left and followed the pattern: day 1, L/L/R; day 2, L/R/R; day 3, L/R/L (and tested on the R). This approach was applied to all peas across experimental groups. As in the first experiment, each training day consisted of three 2-h training sessions separated by 1-h intervals, during which all seedlings were kept in darkness and undisturbed in their individual mazes. During the training phase, seedlings grew and approached the Y-bifurcation of the maze. As described above, all seedlings were checked at the end of the training phase to ensure that they had not yet grown into one or the other arm and had grown to be ca. 1 cm away from the bifurcation of the maze.

At the end of the training phase, seedlings within each group were further split into an experimental sub-group (Fig. 1Bii) and a control sub-group (Fig. 1Biii). Control seedlings (Light, n = 10; Light-Dark, n = 13; Dark, n = 10) were left in darkness and undisturbed during the testing day. On the testing day, experimental subgroups of seedlings (Light, n = 18; Light-Dark, n = 17; Dark, n = 15) were kept at their treatment-specific temperature cycles in the dark, while being exposed to the fan blowing for three 90-min sessions separated by a 90-min interval. The fan was positioned on the opposite arm of the maze where each pea experienced the light (and the fan) during its last training session. The position of the fan was then maintained fixed for all testing sessions. The morning after the testing day, we inspected all seedlings and recorded which arm of the maze they grew into, scoring whether they made the correct (i.e. towards the fan) or incorrect choice (i.e. away from the fan). Once scored, seedlings were transferred from the controlled-environment room, transplanted into larger pots and later replanted in a community garden.

### Phase of the circadian rhythm

The seedlings were initially entrained by a light-dark cycle in conjunction with a temperature cycle. The latter was maintained as sole Zeitgeber during the training and testing phase. Temperature cycle has been previously shown to effectively synchronize circadian rhythms by inducing circadian-type oscillations in the level of RNAs in pea seedlings^16^. The application of three one-hour light pulses during the training days at different phases of the circadian cycle may have induced phase shifts^40^,^41^. Therefore, the phase of the circadian rhythm may have somewhat deviated from the Zeitgeber cycle.

### Statistics

Statistics were based on comparisons between experimental groups (exposure to fan) and control groups (no fan). Since the behaviour of the controls of Experiment 1 and of the ‘Light Group’ of Experiment 2 was determined by last light exposure (phototropism), the question was whether the fan-exposed experimental group could counteract the phototropic effect. The two-tailed Fisher’s Exact Test of independence was used to assess significance.

## Additional Information

**How to cite this article**: Gagliano, M. *et al*. Learning by Association in Plants. *Sci. Rep.* **6**, 38427; doi: 10.1038/srep38427 (2016).

**Publisher's note:** Springer Nature remains neutral with regard to jurisdictional claims in published maps and institutional affiliations.

## Supplementary Material

Supplementary Information

Supplementary Video 1

Supplementary Dataset 1

## Figures and Tables

**Figure 2:**
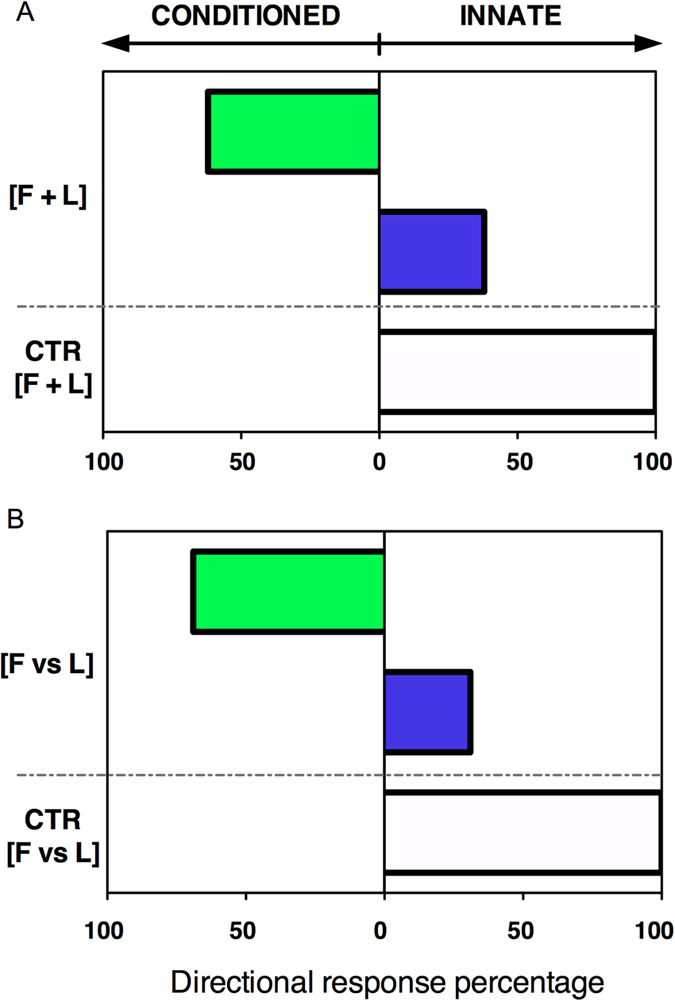
Associative learning in pea seedlings. In the absence of the fan, all control seedlings (100%) directed their growth toward the arm of the maze where the light was last presented (white bars). In the presence of the fan, the majority of seedlings grew toward the arm of the maze that had been associated with light during training ([F + L]: same side; [F *vs* L]: opposite side), thus exhibiting the *conditioned* response (green bars). A smaller proportion of seedlings did not show learning, thus exhibiting the *innate* response (blue bars). The response of the experimental groups was significantly different from controls (Two-tailed Fisher’s Exact Test, P = 0.0027 for [F + L] and P = 0.0017 for [F *vs* L]). See Data file in Supplementary Information.
