# Supplementary material for: Learning by Association in Plants
Source: Sci Rep. 2016 Dec 2;6:38427. doi: 10.1038/srep38427 (PMC5133544; doi:10.1038/srep38427)
Supplement: Supplementary Information [file srep38427-s1.pdf]

Supplementary Information for

Learning by Association in Plants

Monica Gagliano, Vladyslav V. Vyazovskiy, Alexander A. Borbély, Mavra Grimonprez, Martial Depczynski

correspondence to: monica.gagliano@uwa.edu.au

This PDF file includes:

Supplementary Fig. 1 - 2
Caption for Supplementary Video 1

Other Supplementary Materials for this manuscript includes the following:

Supplementary Video 1 (as a separate Video file)
Data File (as a separate Excel file)

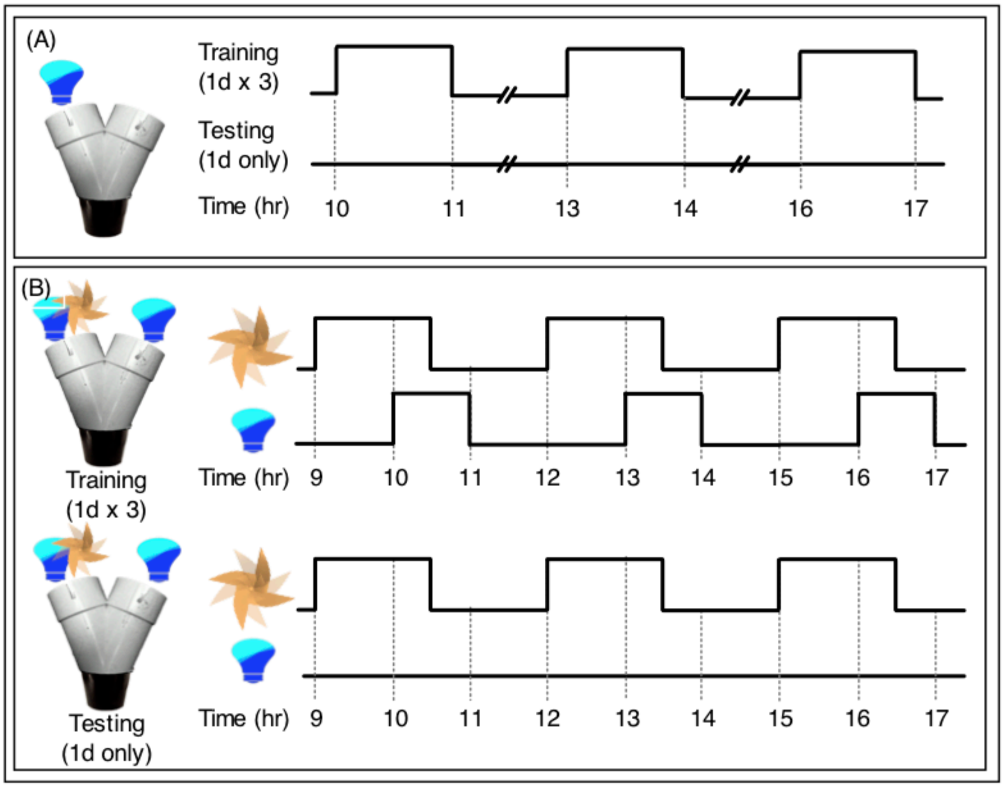

Supplementary Fig. 1. Pilot experiments. The daily protocol of three day training followed by one day testing of pea seedlings to establish the suitability of (A) blue LED light as a unconditioned stimulus, US and (B) airflow produced by a fan as a conditioned stimulus, CS.

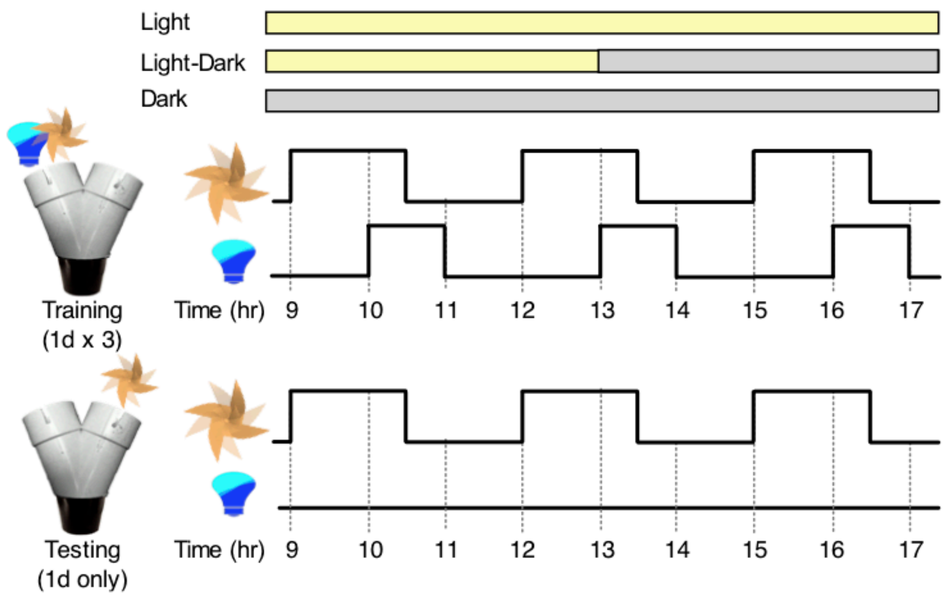

Supplementary Fig. 2. The protocol of the circadian experiment. Training and testing sessions in the 3 groups were positioned within the previous light phase (Light; yellow bar), across the light-dark transition (Light-Dark), and within the previous dark phase (Dark; grey bar) of the 24-h cycle respectively. The sessions occurred at fixed clock times in plants that had been entrained by different phase-shifted Zeitgebers. Training sessions were performed with the fan as the conditioned stimulus, and light as the unconditioned stimulus, on the same arm of the Y-maze. Testing sessions were performed with the fan only.

Supplementary Video 1. A pea seedling undergoing day 1 training in a [F *vs* L] maze. Seedling was filmed during the 30 min period when the fan exposure as the CS on the left arm of the maze overlapped with the delivery of blue light as the US on the opposite arm.
